# Mr. Snow, on the Cure of Agues

**Published:** 1804-10-01

**Authors:** B. G. Snow

**Affiliations:** Woodbridge Barracks, Suffolk


### *To the Editors of the Medical and Phyſical Journal.*

GENTLEMEN,

I Consider it a duty in every practitioner to make as public as possible any circumstance that experience may have thrown in his way that may tend to the alleviation of the calamities of the human race, even in the slightest degree; and under this impression, I am induced to offer you a trifling remedy for the cure of agues, which I have proved to be extremely successful. I am attached to a regiment here; and on my arrival in the latter end of May, found seven or eight of our soldiers labouring under obstinate intermittents: every remedy that is usually prescribed for such cases, had been exhibited without effect. I recommended that each man should take half a grain of tartar antim. the moment he felt the paroxysm approaching, and repeat the dose in about ten minutes or a quarter of hour hour; it generally produces only nausea, but sometimes vomiting; it lessens immediately the duration of the fit, and its severity, and the patients express a great wish that the same medicine may be again prescribed them: in a week or ten days the whole were completely cured.

His Majesty's 24th regiment came into these barracks about six weeks since, and among the number of their sick were two of agues, of long standing. I proposed to Mr. Featherstone, the surgeon, to allow me to give them the antim. tart. which he very liberally and readily consented to, observing, that he had been giving them the arsenical solution, bark, and every thing he could think of, without effect: These men had no symptoms whatever of ague at the expiration of five days.

In a voyage I once made to India, I had an ample opportunity of proving the good effect of producing sickness in this disease. Our ship was lying in Whampoo River, in China, during the time the Paddy-fields were overflowed, and agues in a short time became so very prevalent among the crews of the different Indiamen, that some of them had not men left sufficiently healthy to perform the regular duty of the ship. The ship of which I was surgeon (the Caledonian) was nearly in that state; my practice at that time was to give emetics previous to the accession of the paroxysm, and I found no difficulty in curing very soon every case that came under my care. But I have every reason to believe that vomiting is not at all necessary to the cure; that nausea is alone sufficient; and I beg to ask, may not the good effect produced by arsenic in this disease arise purely from the sickness it causes, and not from any tonic power it possesses?

I am, &c.

*Woodbridge Barracks, Suffolk,*
*September* 9, 1804.

B. G. SNOW.

